# Nuclear EGFR signalling network in cancers: linking EGFR pathway to cell cycle progression, nitric oxide pathway and patient survival

**DOI:** 10.1038/sj.bjc.6602941

**Published:** 2006-01-24

**Authors:** H-W Lo, M-C Hung

**Affiliations:** 1 Department of Molecular and Cellular Oncology, University of Texas MD Anderson Cancer Center, 1515 Holcombe Blvd, Houston, TX 77030, USA

**Keywords:** epidermal growth factor receptor, inducible nitric oxide synthase, transcriptional regulation, cell cycle control, receptor tyrosine kinase

## Abstract

Emerging evidences suggest the existence of a new mode of epidermal growth factor receptor (EGFR) signalling pathway in which activated EGFR undergoes nuclear translocalization and subsequently regulates gene expression and potentially mediates other cellular processes. This signalling route is distinct from the better-characterized, traditional EGFR pathway that involves transduction of mitogenic signals through activation of multiple signalling cascades. Transcriptional activity of nuclear EGFR appears to depend on its C-terminal transactivation domain and its physical and functional interaction with other transcription factors that contain DNA-binding activity. Likely via its ability to upregulate gene expression, nuclear EGFR pathway is associated with major characteristics of more aggressive tumours: increased proliferative potential, nitric oxide synthesis, and accelerated G1/S cell cycle progression. A role of nuclear EGFR in prognostic prediction is further suggested in patients with breast carcinomas and oropharyngeal squamous cell carcinomas. It is noted that significant advances were made towards the knowledge of the nuclear EGFR pathway; however, many aspects of this new pathway remain unresolved and will be discussed in this review. As a number of other receptor tyrosine kinases (RTKs) and cytokine receptors also undergo similar nuclear translocalization, a better understanding of the physiological and malignant nature of the nuclear EGFR pathway will likely shed light into the biology of cancer with nuclear RTKs.

Albeit very little is known about physiological function and cancer relevance of the nuclear EGFR pathway until recent years, EGFR has been consistently detected in the nuclei of cancer cells and primary tumour specimens of various origins as well as in those of other highly proliferative tissues (Marti *et al*, 1991; Cao *et al*, 1995; Lin *et al*, 2001; Lo *et al*, 2005a, 2005b). Increased expression of nuclear EGFR is linked to poor clinical outcome in patients with breast carcinomas (Lo *et al*, 2005c) and oropharyngeal squamous cell carcinomas (Psyrri *et al*, 2005). Consistently, nuclear accumulation of EGFR correlates with increased expression of cyclin D1, inducible nitric oxide synthase (iNOS) and B-Myb, all of which are frequently overexpressed in human cancers and associated with increased cell proliferation (Lin *et al*, 2001; Hanada *et al*, 2005; Lo *et al*, 2005a). Furthermore, most recent reports indicate a plausible mechanism underlying nuclear EGFR-mediated gene regulation, which involves a physical interaction with other transcription factors, signal transducer and activator of transcription 3 (STAT3) and E2F1 (Hanada *et al*, 2005; Lo *et al*, 2005a). A cellular mechanism that can potentially account for nuclear import of receptor tyrosine kinases (RTKs) is also proposed recently (Giri *et al*, 2005). It is important to note that nuclear import is not only observed with EGFR but also with many other RTKs, including mouse erbb1, HER-2, rat p185neu, HER-3, truncated C-terminal HER-4, and fibroblast growth factor receptor (FGFR) and cytokine receptors (Marti *et al*, 1991; Xie and Hung, 1994; Lin *et al*, 2001; Ni *et al*, 2001; Offterdinger *et al*, 2002; Schausberger *et al*, 2003; Wang *et al*, 2004; Krolewski, 2005). As RTK-mediated pathways are frequently deregulated in many human cancers and are closely linked to tumorigenesis and tumour progression, it is thus an urgent task to better understand the nature of these nuclear RTKs and, more importantly, to determine the extent to which they contribute to the malignant biology and therapeutic response of human cancers.

## DETECTION OF NUCLEAR EGFR

Nuclear detection of EGFR was first reported in hepatocytes that underwent regeneration and in primary adrenocortical carcinomas more than a decade ago (Kamio *et al*, 1990; Marti *et al*, 1991). Nuclear expression of EGFR was further detected in other cell types and tissues, such as placentas, thyroids and immortalized epithelial cells of ovary and kidney origins (Cao *et al*, 1995; Lin *et al*, 2001; Marti *et al*, 2001; Lo *et al*, 2005a). High levels of EGFR was also found in the nuclei of many tumours, including those of skin, breast, bladder, cervix, adrenocorticord, thyroid and oral cavity (Kamio *et al*, 1990; Lipponen and Eskelinen, 1994; Lin *et al*, 2001; Marti *et al*, 2001; Lo *et al*, 2005a, 2005c; Psyrri *et al*, 2005). In addition, EGFR has been shown to localize in the inner nuclear membrane (Cao *et al*, 1995; Klein *et al*, 2004). Nuclear counterpart of EGFR appears to be the full-length receptor and likely, in the phosphorylated form, as shown by a number of studies (Cao *et al*, 1995; Lin *et al*, 2001; Cordero *et al*, 2002; Dittmann *et al*, 2005a; Lo *et al*, 2005a, 2005c). Consistently, EGF and pro-TGF-*α* were found to translocate into the nucleus of proliferating hepatocytes (Raper *et al*, 1987; Schausberger *et al*, 2003). In addition to ligand stimulation, nuclear translocalization of EGFR can be initiated by irradiation, heat shock, H_2_O_2_ and cisplatin (Cao *et al*, 1995; Lin *et al*, 2001; Dittmann *et al*, 2005a). Conversely, EGF- and irradiation-induced EGFR nuclear transport can be blocked by 1,25-dihydroxyvitamin D (Cordero *et al*, 2002) and anti-EGFR antibody, C225/Cetuximab (Dittmann *et al*, 2005b), respectively.

## NUCLEAR EXISTENCE OF OTHER RTKS

Nuclear translocalization is not a unique event with EGFR but rather a universal phenomenon occurring to many other cell-surface receptors. In addition to EGFR and its mouse homologue erbb1, all other receptors in the ErbB family have also been detected in the nucleus (Xie and Hung, 1994; Lin *et al*, 2001; Ni *et al*, 2001; Offterdinger *et al*, 2002; Schausberger *et al*, 2003; Wang *et al*, 2004; Krolewski, 2005). Rat p185neu, HER-2 and HER-3 receptors exist as full-length receptors in cell nucleus (Xie and Hung, 1994; Offterdinger *et al*, 2002; Wang *et al*, 2004) whereas HER-4 undergoes *γ*-secretase-mediated cleavage and the C-terminal 80-kDa fragment translocates into the nucleus (Ni *et al*, 2001). Several other RTKs, including TrkA,B/NGFR, FGFR, VEGF receptor 2 (VEGFR-2) and type I TGF-*β* receptor also undergo nuclear transport (Rakowicz-Szulczynska *et al*, 1988; Maher, 1996; Zwaagstra *et al*, 2000; Pillai *et al*, 2005). Inflammatory cytokine receptors, such as, those of interleukin-1 (IL-1), IL-5 and interferon-*γ* (IFN-*γ*) also exist in the nuclear compartment (Curtis *et al*, 1990; Jans and Hassan, 1998; Larkin *et al*, 2000). In line with these observations, ligands to most of these receptors were also found in the nucleus (Raper *et al*, 1987; Curtis *et al*, 1990; Jans and Hassan, 1998; Schausberger *et al*, 2003).

### POTENTIAL MECHANISMS FOR NUCLEAR–CYTOPLASMIC TRANSPORT OF CELL-SURFACE RECEPTORS

**Figure 1:**
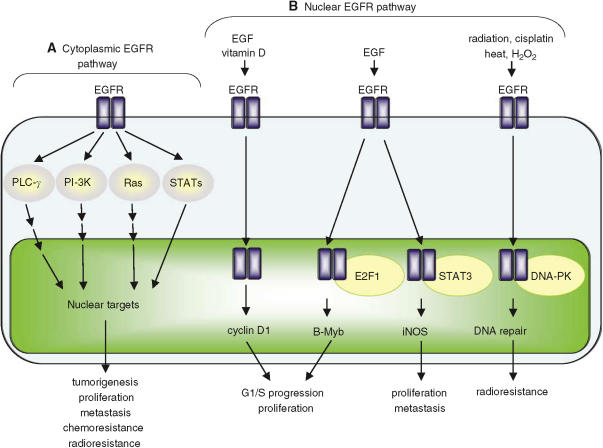
Cytoplasmic/traditional and nuclear modes of the EGFR signalling pathway. The EGFR signalling pathway exerts its biological effects via two major modes of actions, namely, cytoplasmic/traditional (**A**) and nuclear (**B**) modes. (**A**) The cytoplasmic EGFR pathway is consisted of four major modules: PLC-*γ*-CaMK/PKC, Ras-Raf-MAPK, PI-3K-Akt-GSK and STATs. Activation of these signalling modules often leads to tumorigenesis, tumour proliferation, metastasis, chemoresistance and radioresistance. (**B**) The nuclear EGFR pathway can be initiated by ligand binding and exposure to vitamin D, radiation, cisplatin, heat and H_2_O_2._ Following nuclear translocalization, nuclear EGFR interacts with DNA-binding transcription factors, E2F1 and STAT3, and activates expression of B-Myb and iNOS, respectively. Nuclear EGFR also upregulates cyclin D1 gene expression. Increased expression of cyclin D1 and B-Myb contributes to accelerated G1/S cell cycle progression and, on the other hand, elevated iNOS is associated with tumour proliferation and metastasis. Upon DNA damage and oxidative/heat stress, EGFR enters the cell nucleus and interacts with DNA-PK, leading to DNA repair and radioresistance.

Several lines of evidences suggest that many nuclear RTKs can be originated from the cell-surface counterparts and exist as un-cleaved full-length receptors, including EGFR, HER-2, HER-3, and FGFR1 (Maher, 1996; Lin *et al*, 2001; Offterdinger *et al*, 2002; Wang *et al*, 2004; Dittmann *et al*, 2005a; Giri *et al*, 2005; Lo *et al*, 2005c) (Figure 1). VEGFR2 (Shay-Salit *et al*, 2002), IFN*γ*R-1, IFN*γ*R-2 (Jans and Hassan, 1998; Larkin *et al*, 2000), IL-1R (Curtis *et al*, 1990) also localize in the nucleus as intact receptors. Spliced variants of cell-surface FGFR1 and the intracellular domain of HER-4 have been found to enter the cell nucleus (Stachowiak *et al*, 1997; Ni *et al*, 2001, 2003); however, it is yet clear whether TrkA, TrkB (Zhang *et al*, 2003), type I TGF-*β* receptor (Zwaagstra *et al*, 2000) and IL-5R (Jans *et al*, 1997) undergo nuclear translocalization as full-length receptors. While the mechanisms underlying nuclear import of the intracellular domain of HER-4 are better-characterized (Ni *et al*, 2001, 2003), those account for full-length receptors remain less clear as they are complicated by the membrane-associated nature of the full-length receptors.

Nuclear import of cell-surface receptors has been shown to occur in ligand-dependent and -independent manners. Ligand stimulation activates nuclear translocalization of many cell-surface receptors including EGFR, FGFR, IFN-*γ*R, IL-1R, type I TGF-*β* receptor and IL-5R (Curtis *et al*, 1990; Jans and Hassan, 1998; Larkin *et al*, 2000; Zwaagstra *et al*, 2000; Lin *et al*, 2001). In the case of HER-2 which lacks the domain/ability for ligand-binding, its kinase activity is required for nuclear entry (Wang *et al*, 2004). Nuclear import of EGFR and type I TGF-*β* receptor can occur in a ligand-independent manner (Zwaagstra *et al*, 2000; Dittmann *et al*, 2005a). In contrast, heregulin *β*1 activates nuclear export of HER-3 in mammary epithelial cells, which contain high levels of nuclear HER-3 under normal growth conditions (Offterdinger *et al*, 2002).

For those cell-surface receptors that undergo nuclear import following ligand binding, it is speculated and supported by several recent reports that receptor internalization may serve as an initial step for its transit from the cell-surface to the nucleus, as ligand activation is coupled with receptor internalization (Bryant and Stow, 2005). Blocking receptor internalization/endocytosis using Dynamin II mutant, Dynamin II K44A, prevents nuclear import of EGFR, HER-2 and FGFR (Bryant *et al*, 2005; Giri *et al*, 2005; Lo *et al*). However, it is yet clear with regard to the individual contribution of clathrin-, lipid raft- and caveolin-dependent endocytosis to this process. Several lines of evidences further suggest the possibility that endocytic sorting machinery may be utilized to shuttle internalized receptors to the perinuclear and nuclear regions (Bryant *et al*, 2005; Giri *et al*, 2005).

In the light of the observation that many cell-surface receptors with nuclear–cytoplasmic shuttling appear to exist as non-membrane-bound receptors in the nucleus, it is thus speculated that cells may utilize a general mechanism for extracting these transmembrane receptors prior to its passage through the nuclear pore complex (NPC). However, such mechanism has not yet identified. Nevertheless, it is becoming clear that several cell-surface receptors are capable of interacting with nuclear transport receptors, importins *α*/*β* and exportins, and thus enter and exit the cell nucleus, respectively (Reilly and Maher, 2001; Giri *et al*, 2005). Association with importin *β*1 is important for nuclear import of HER-2 and FGFR (Reilly and Maher, 2001; Giri *et al*, 2005) whereas EGFR has been shown to interacts with both importin *β*1 and importin *α* (Dittmann *et al*, 2005a; Lo *et al*). Blockage of RanGDP and importin *β*1 by dominant-negative mutant and siRNA, respectively, also inhibits nuclear transport of HER-2, suggesting that NPC is involved in the process (Giri *et al*, 2005).

Nuclear localization signals (NLSs) within EGFR, HER-2, HER-3 and HER-4 have been identified (Offterdinger *et al*, 2002; Wang *et al*, 2004; Williams *et al*, 2004; Lo *et al*, 2005a). Interestingly, EGFR, HER-2 and HER-4 contain their NLSs within the juxtamembrane region whereas HER-3′s NLS is located in the C-terminal region. On the other hand, very little is known about the nuclear export process. Recent evidences suggest that nuclear export of EGFR, HER-2 and HER-3 may involve exportin CRM1 (Offterdinger *et al*, 2002; Giri *et al*, 2005; Lo *et al*). Existence of nuclear export sequences within these cell-surface receptors, however, has not been demonstrated.

### NUCLEAR EGFR AND HER-2 AS TRANSCRIPTIONAL REGULATOR

A role of nuclear ErbBs in transcriptional regulation was first shown by the observation that cytoplasmic domain of rat p185neu contains transactivational activity when tested in a GAL4-reporter system (Xie and Hung, 1994). More recently, transactivational domains within EGFR, HER-2 and HER-4 were also found to be functional (Lin *et al*, 2001; Ni *et al*, 2001; Wang *et al*, 2004). Using unbiased approaches, nuclear EGFR and HER-2 were further shown to associate with specific DNA sequences designated AT-rich sequence (ATRS) and HER-2-associated sequence (HAS), respectively (Lin *et al*, 2001; Wang *et al*, 2004). Promoters that are targeted by nuclear EGFR are those of cyclin D1, iNOS and B-Myb (Lin *et al*, 2001; Hanada *et al*, 2005; Lo *et al*, 2005a). Nuclear HER-2 binds to promoters of cyclooxygenase-2 (COX-2), PRPK, MMP-16 and DDX-10, whereas nuclear HER-4 associates with that of *β*-casein (Wang *et al*, 2004; Williams *et al*, 2004).

Given the notion that ErbB receptors lack a putative DNA-binding domain, it is suspected that these receptors first associate with DNA-binding transcription factors and then enhance target gene transcription via their intrinsic transactivational activity. In this regard, nuclear EGFR interacts with STAT3 and coregulates iNOS expression (Lo *et al*, 2005a). Nuclear EGFR/E2F1 complex activates expression of B-Myb, a positive regulator of G1/S cell cycle progression (Hanada *et al*, 2005). Nuclear HER-4 forms a complex with STAT5a and coactivates *β*-casein gene promoter (Williams *et al*, 2004). However, it is suggested that HER-4 may have a weak transactivational activity and requires interaction with a strong transcription coactivator YAP for its gene regulatory function (Komuro *et al*, 2003). In line with this observation, nuclear FGFR associates with and activates transcription coactivator CBP to upregulate gene promoters (Fang *et al*, 2005). Known targets of nuclear FGFR include FGF-2, neurofilament-L and tyrosine hydroxylase (Peng *et al*, 2001, 2002; Stachowiak *et al*, 2003). In support of this notion, EGF, EGFR, TrkA/NGF receptor and the ligand NGF have been shown to bind to chromatins (Rakowicz-Szulczynska *et al*, 1988; Kamio *et al*, 1990) and Schwannoma-derived growth factor, a ligand for EGFR, bound to A+T-rich DNA sequences (Kimura, 1993). Together, these data suggest an emerging role that nuclear receptors play in transcriptional regulation.

## LINKING NUCLEAR EGFR TO PATHWAYS THAT ARE IMPORTANT FOR TUMOUR BIOLOGY

Transcriptional targets of nuclear EGFR and HER-2, those identified thus far, are closely involved in tumorigenesis, and tumour proliferation and progression (Lin *et al*, 2001; Wang *et al*, 2004; Hanada *et al*, 2005; Lo *et al*, 2005a). Both cyclin D1 and B-Myb are positive regulators of G1/S progression (Lin *et al*, 2001; Joaquin and Watson, 2003). COX-2 and iNOS are enzymes that produce prostagladins and nitric oxide, respectively, and emerge as major targets for chemoprevention and chemotherapy (Gupta and Dubois, 2001; Xu *et al*, 2002). Consistently, a positive correlation has been found between nuclear EGFR and cyclin D1/iNOS in a cohort of breast carcinomas (Lo *et al*, 2005a, 2005c). In the same tumour cohort, high levels of nuclear HER-2 associate with COX-2 overexpression (Wang *et al*, 2004). Furthermore, nuclear expression of EGFR positively correlates with that of Ki-67, an indicator of active proliferation (Lo *et al*, 2005c). A casual correlation of nuclear accumulation of EGFR/mouse erbb1 and their ligands EGF/TGF-*α* with cell proliferation/DNA synthesis has been reported by several studies (Marti *et al*, 1991; Schausberger *et al*, 2003). In agreement with its role in proliferation/DNA synthesis, EGFR undergoes nuclear translocalization in regenerating livers (Marti *et al*, 1991), pregnant uterus and proliferative basal cells within normal mouth mucosa (Lin *et al*, 2001). A potential role of nuclear EGFR in DNA damage/repair in response to irradiation/oxidative stress has also been suggested (Dittmann *et al*, 2005a, 2005b).

Furthermore, nuclear import of FGFR is associated with proliferation (Reilly and Maher, 2001), which is in line with the observation that nuclear FGFR enhances c-jun expression (Reilly and Maher, 2001). Also suggested by accumulating evidences is that nuclear FGFR mediates cAMP-activated expression of neurofilament-L and, thus, is important for cAMP-induced differentiation of neuronal progenitor cells into neurons (Stachowiak *et al*, 2003).

While the pathological significance of nuclear RTKs remains elusive, two recent reports suggest the potential use of nuclear EGFR as a prognostic indicator for poor clinical outcome (Lo *et al*, 2005c; Psyrri *et al*, 2005). In a cohort of 130 breast carcinomas, an inverse correlation was found between nuclear EGFR, but not the non-nuclear counterpart, and overall patient survival (Lo *et al*, 2005c). This observation is consistent with the notion that total EGFR levels serve as a moderate prognostic indicator in breast cancer patients and thus suggests a novel prognostic value of nuclear EGFR in these patients. A positive association was also observed between non-nuclear/nuclear EGFR and overall survival of patients with oral squamous cell carcinomas (Lo *et al*, 2005c). Using a cohort of 95 patients with oropharyngeal squamous cell carcinomas, Psyrri *et al* (2005) found that both total and nuclear EGFR levels predict poor clinical outcomes as measured by local recurrence and poor disease-free survival. However, no significant correlation was found between EGFR, total and nuclear expression, and overall survival rates in these patients (Psyrri *et al*, 2005). Despite with relatively small cohorts, these studies provided rationales for future extensive research that examines the prognostic value of EGFR in larger population with various cancer types. This task is particularly important and of cancer-relevance because of the following reasons: (i) the EGFR signalling pathway is highly de-regulated in many human cancers and is an attractive target for anti-tumour therapy, (ii) anti-EGFR therapy are only effective to certain patients, (iii) a correlation is often lacking between EGFR expression and tumour responsiveness to anti-EGFR treatments, and (iv) previously overlooked nuclear EGFR pathway is linked to aggressive tumour biology.

Moreover, in the light of the report showing that 1,25-dihydroxyvitamin D inhibited EGF-induced EGFR nuclear transport, vitamin D1 may in part exert its anticancer effect via blocking the nuclear EGFR pathway (Cordero *et al*, 2002). Similarly, anti-EGFR antibody (C225/Cetuximab) blocks radiation-induced nuclear EGFR transport and its interaction with DNA-dependent protein kinase, an enzyme involved in DNA-repair, and thus may lead to radiosensitization (Dittmann *et al*, 2005a, 2005b). Interestingly, in women with breast cancer, nuclear HER-4 has been recently shown to associate with poor survival compared to those who had membrane HER-4 expression (Junttila *et al*, 2005). Together, these reports suggest that nuclear EGFR may play a potential important role in the aggressive biology of cancers, radiosensitivity and clinical outcomes.

## CONCLUSIONS

Accumulating reports reveal a new EGFR signalling pathway that escapes the traditional transduction cascades but involves direct shuttling of activated EGFR into the cell nucleus. Nuclear existence of EGFR has been observed for more than a decade in normal cells undergoing active proliferation and in cancerous cells. The physiological function of nuclear EGFR, however, was not elucidated until recently to involve regulation of gene transcription and possibly other nuclear events. Nuclear EGFR, HER-2, HER-4 and FGFR contain intrinsic ability to enhance gene transcription. In addition to transcriptional regulation, nuclear RTKs may have other functions such as DNA damage/repair. Mechanistic studies further provide plausible mechanisms by which nuclear EGFR/HER-4 turn on gene expression, which involve their intrinsic transactivation domain and physical interaction with other transcription factors that contain DNA-binding domains. STAT3 and E2F1 have been identified as transcription co-factors of nuclear EGFR whereas STAT5a partners with nuclear HER-4. Via the ability of nuclear EGFR to associate with STAT3 and E2F1 and to upregulate expression of cyclin D1, iNOS and B-Myb, a new link is established that associates EGFR with several cellular processes such as cell cycle progression and nitric oxide pathway. Consistent with the role in liver regeneration, nuclear EGFR accumulation is associated with increased proliferation in cancer cells and hepatocytes. Albeit correlative studies suggest an inverse association of high nuclear EGFR and poor clinical outcome, the pathological role of nuclear EGFR remains largely unknown and is in need of further investigations. For instance, it is yet to be determined whether nuclear EGFR plays a crucial role in the genesis, progression, metastatic growth and/or therapeutic responses of human cancers. Also elusive is the pathological involvement of nuclear HER-2, HER-3 and HER-4 in human cancers. All these unaddressed issues will be crucial in advancing our knowledge of the malignant nature of the nuclear RTK pathways. In summary, emerging new evidences provide important insights into a signalling path that has been overlooked for past decades and thus prompt for an urgent need to further unravel the physiological and oncogenic properties of the nuclear EGFR pathway.
